# Supplementary material for: Evolution of fertilization ability in obligatorily outcrossing populations of Caenorhabditis elegans
Source: PeerJ. 2023 Sep 8;11:e15825. doi: 10.7717/peerj.15825 (PMC10494835; doi:10.7717/peerj.15825)
Supplement: Supplemental Information 1 — A–G represents populations from 20 °C, while H–T represents 24 °C. Plots are generated from raw data, where all three replicates are visible and marked with different point shapes. The blue lines were obtained by running a linear model from analysis 1. Results for this model are visible in Table 2. [file peerj-11-15825-s001.pdf]

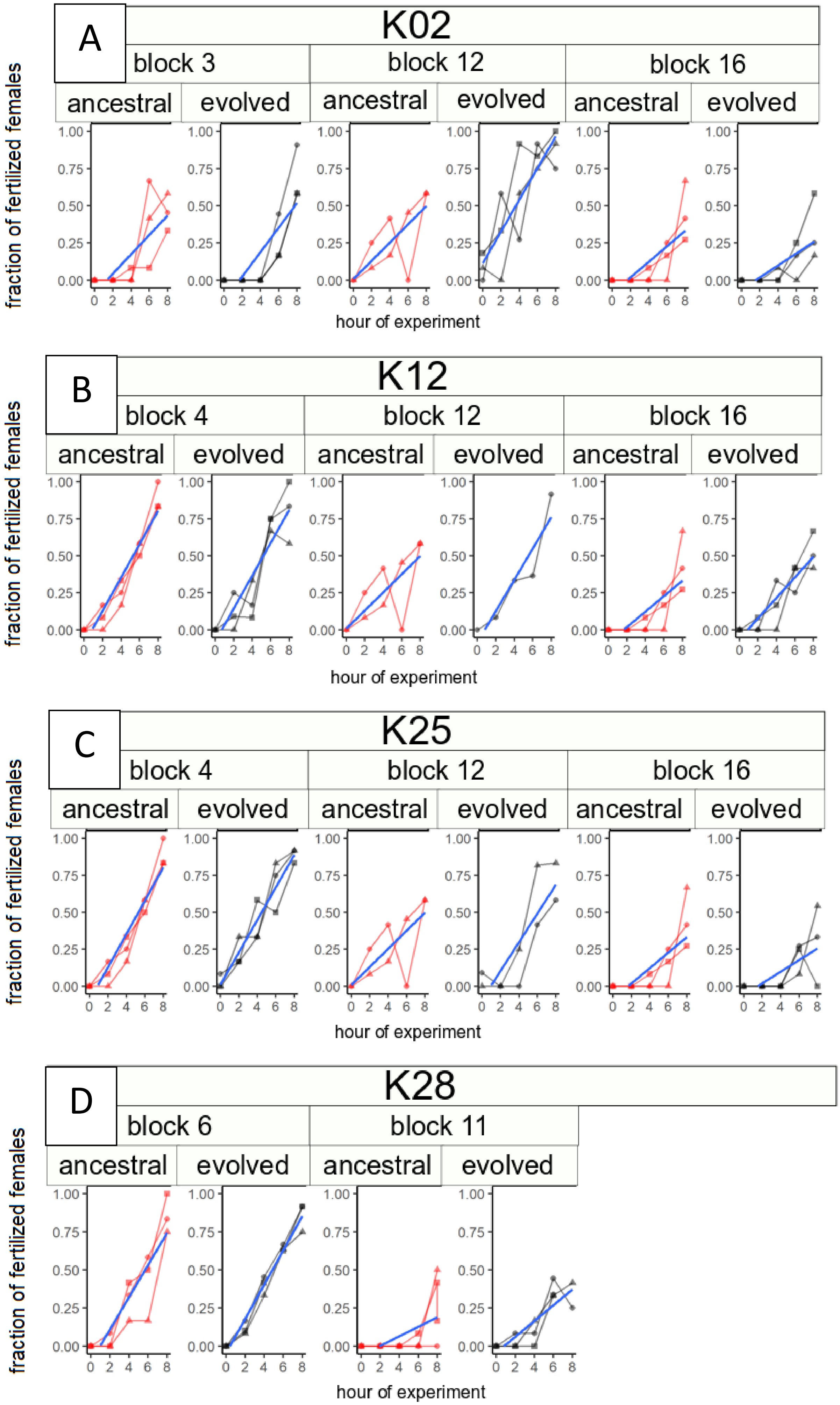

A
K02
block 3
block 12
block 16
ancestral
evolved
fraction of fertilized females
hour of experiment
B
K12
block 4
block 12
block 16
ancestral
evolved
fraction of fertilized females
hour of experiment
C
K25
block 4
block 12
block 16
ancestral
evolved
fraction of fertilized females
hour of experiment
D
K28
block 6
block 11
ancestral
evolved
fraction of fertilized females
hour of experiment

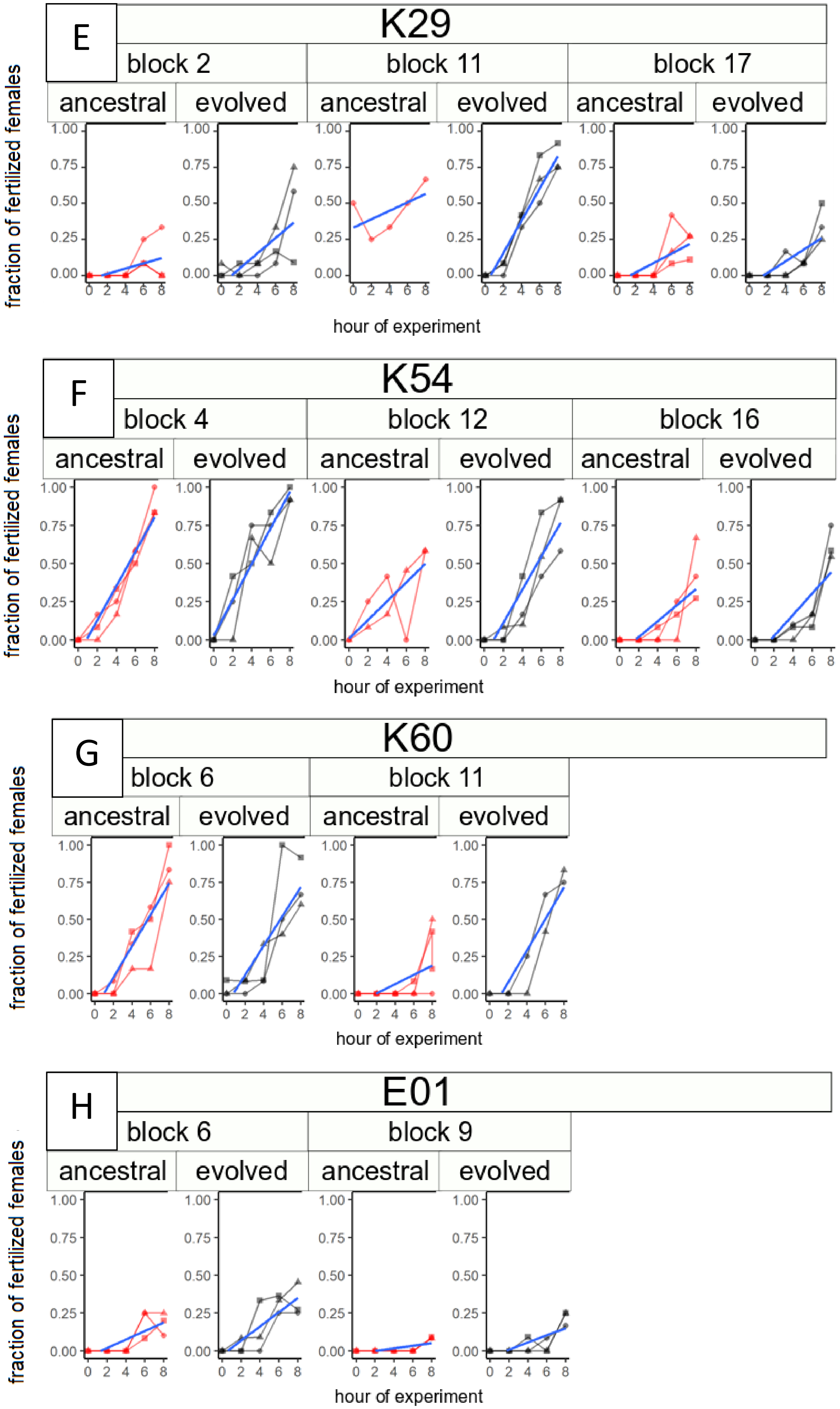
E
K29
block 2
block 11
block 17
ancestral
evolved
fraction of fertilized females
hour of experiment
F
K54
block 4
block 12
block 16
ancestral
evolved
fraction of fertilized females
hour of experiment
G
K60
block 6
block 11
ancestral
evolved
fraction of fertilized females
hour of experiment
H
E01
block 6
block 9
ancestral
evolved
fraction of fertilized females
hour of experiment

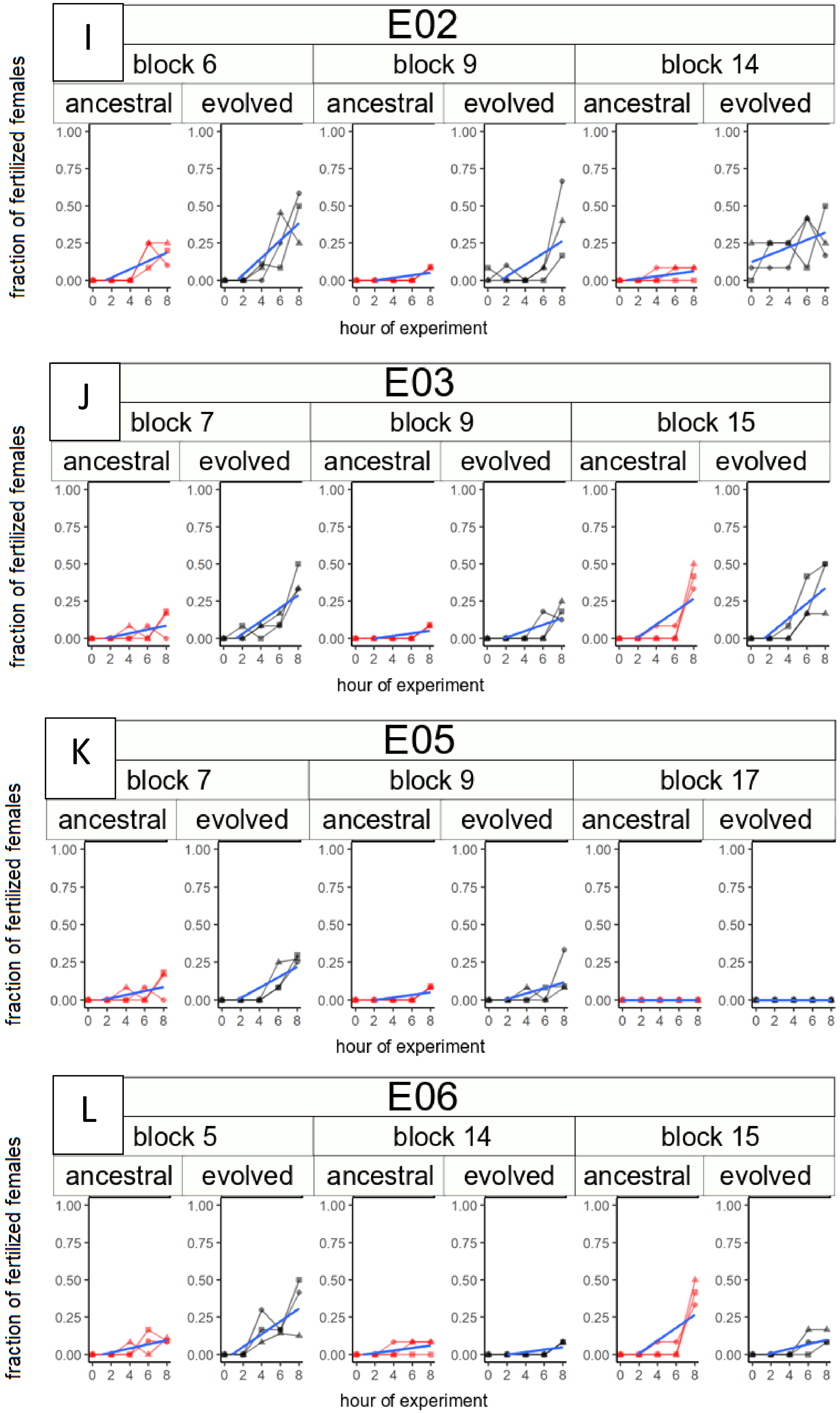
I
E02
block 6
block 9
block 14
ancestral
evolved
fraction of fertilized females
hour of experiment
J
E03
block 7
block 9
block 15
ancestral
evolved
fraction of fertilized females
hour of experiment
K
E05
block 7
block 9
block 17
ancestral
evolved
fraction of fertilized females
hour of experiment
L
E06
block 5
block 14
block 15
ancestral
evolved
fraction of fertilized females
hour of experiment
0.00
0.25
0.50
0.75
1.00
0
2
4
6
8

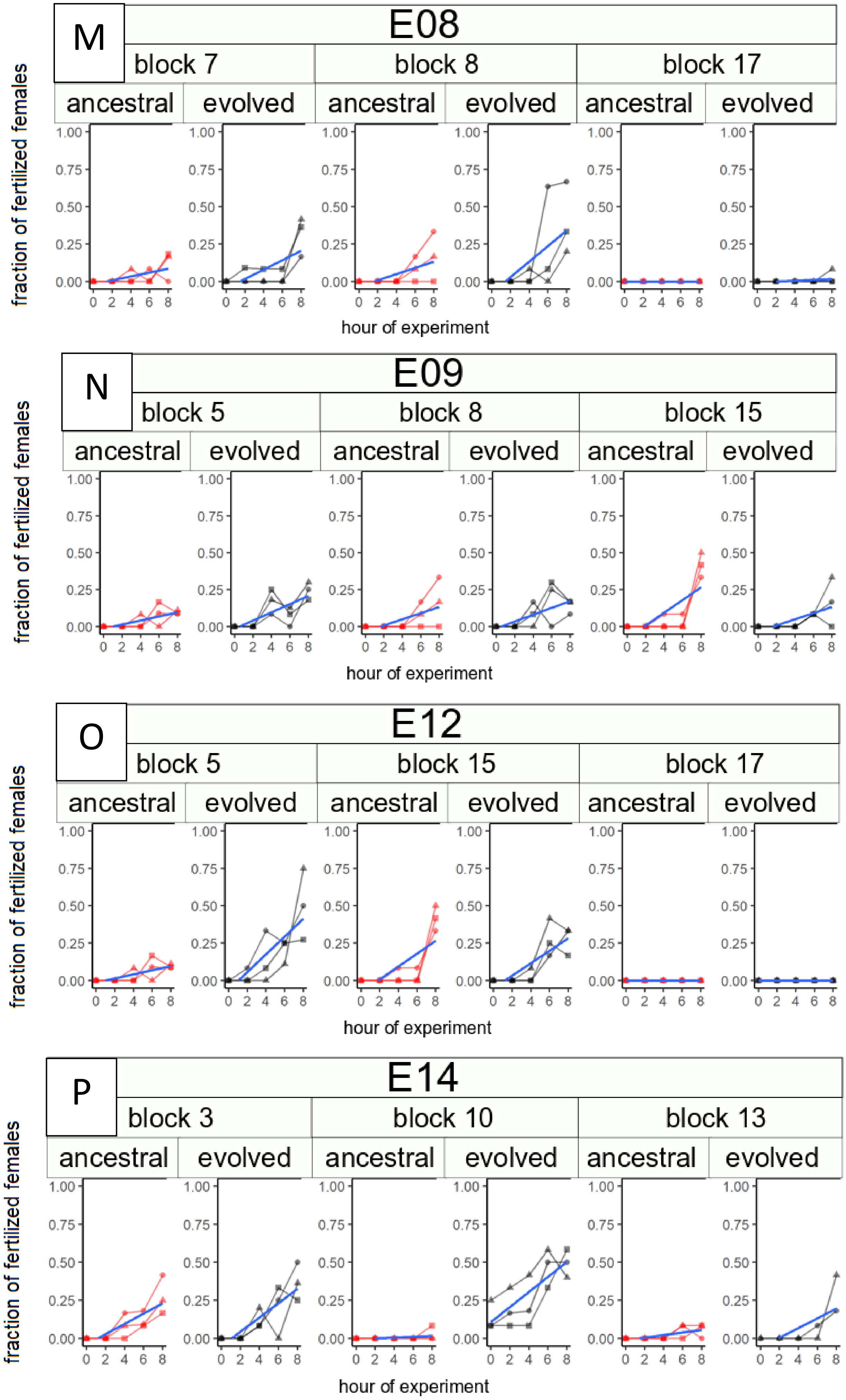

M
E08
block 7
block 8
block 17
ancestral
evolved
fraction of fertilized females
1.00
0.75
0.50
0.25
0.00
0 2 4 6 8
hour of experiment
N
E09
block 5
block 8
block 15
ancestral
evolved
fraction of fertilized females
hour of experiment
O
E12
block 5
block 15
block 17
ancestral
evolved
fraction of fertilized females
hour of experiment
P
E14
block 3
block 10
block 13
ancestral
evolved
fraction of fertilized females
hour of experiment

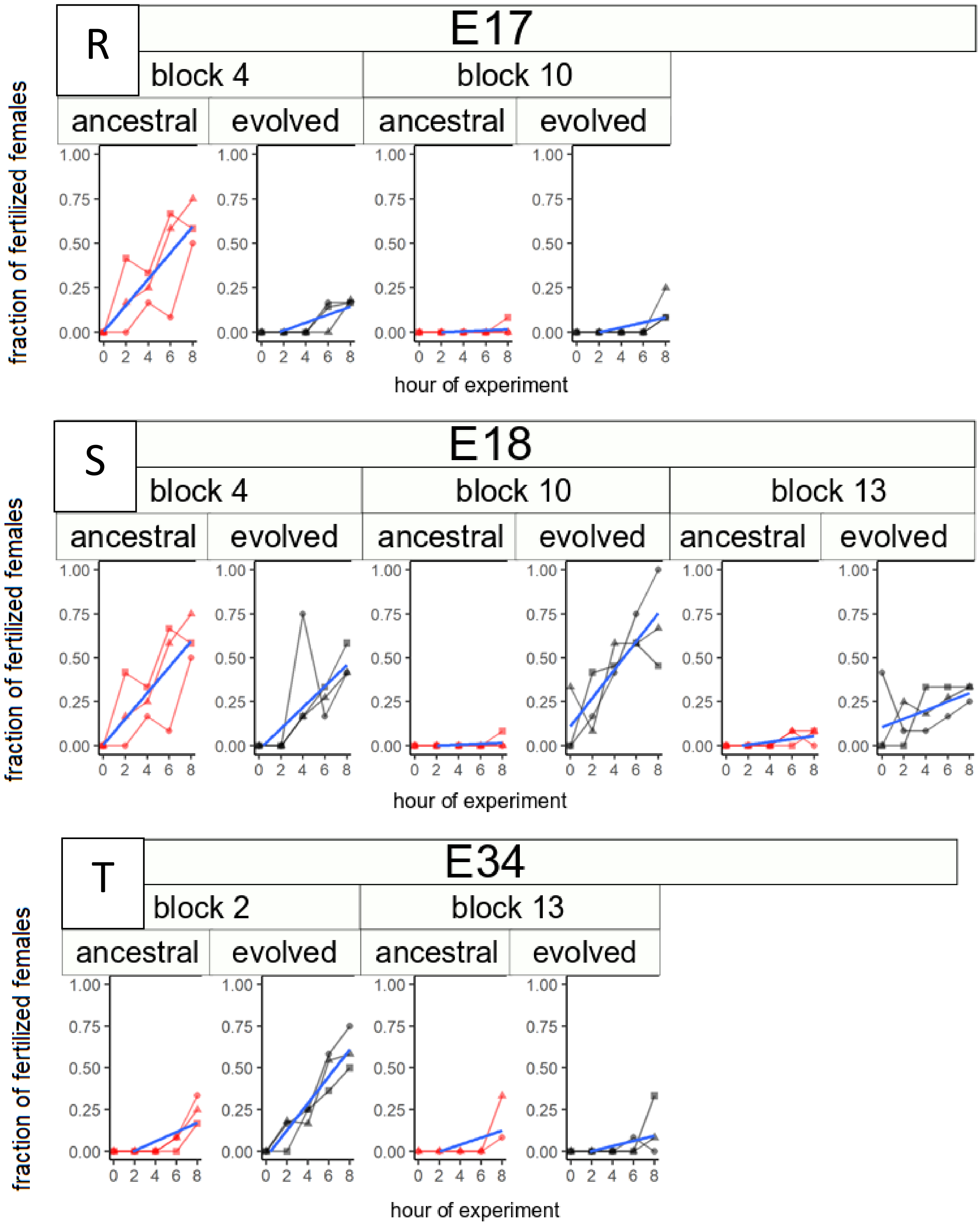

R
E17
block 4
block 10
ancestral
evolved
ancestral
evolved
fraction of fertilized females
1.00
0.75
0.50
0.25
0.00
0 2 4 6 8
hour of experiment
S
E18
block 4
block 10
block 13
ancestral
evolved
ancestral
evolved
ancestral
evolved
fraction of fertilized females
1.00
0.75
0.50
0.25
0.00
0 2 4 6 8
hour of experiment
T
E34
block 2
block 13
ancestral
evolved
ancestral
evolved
fraction of fertilized females
1.00
0.75
0.50
0.25
0.00
0 2 4 6 8
hour of experiment
