# Supplementary figures and images for: Evolution of fertilization ability in obligatorily outcrossing populations of Caenorhabditis elegans
Source: PeerJ. 2023 Sep 8;11:e15825. doi: 10.7717/peerj.15825 (PMC10494835; doi:10.7717/peerj.15825)

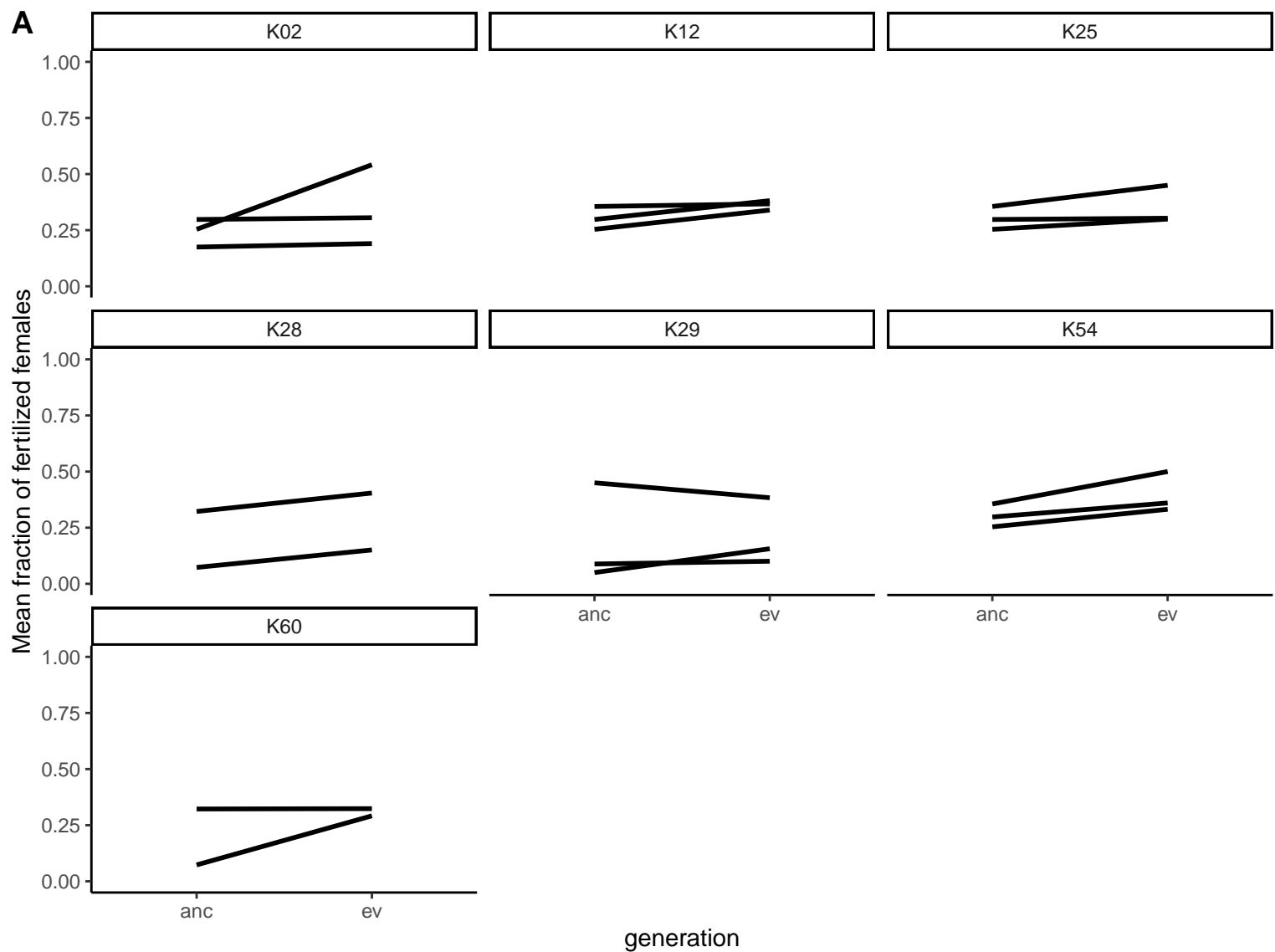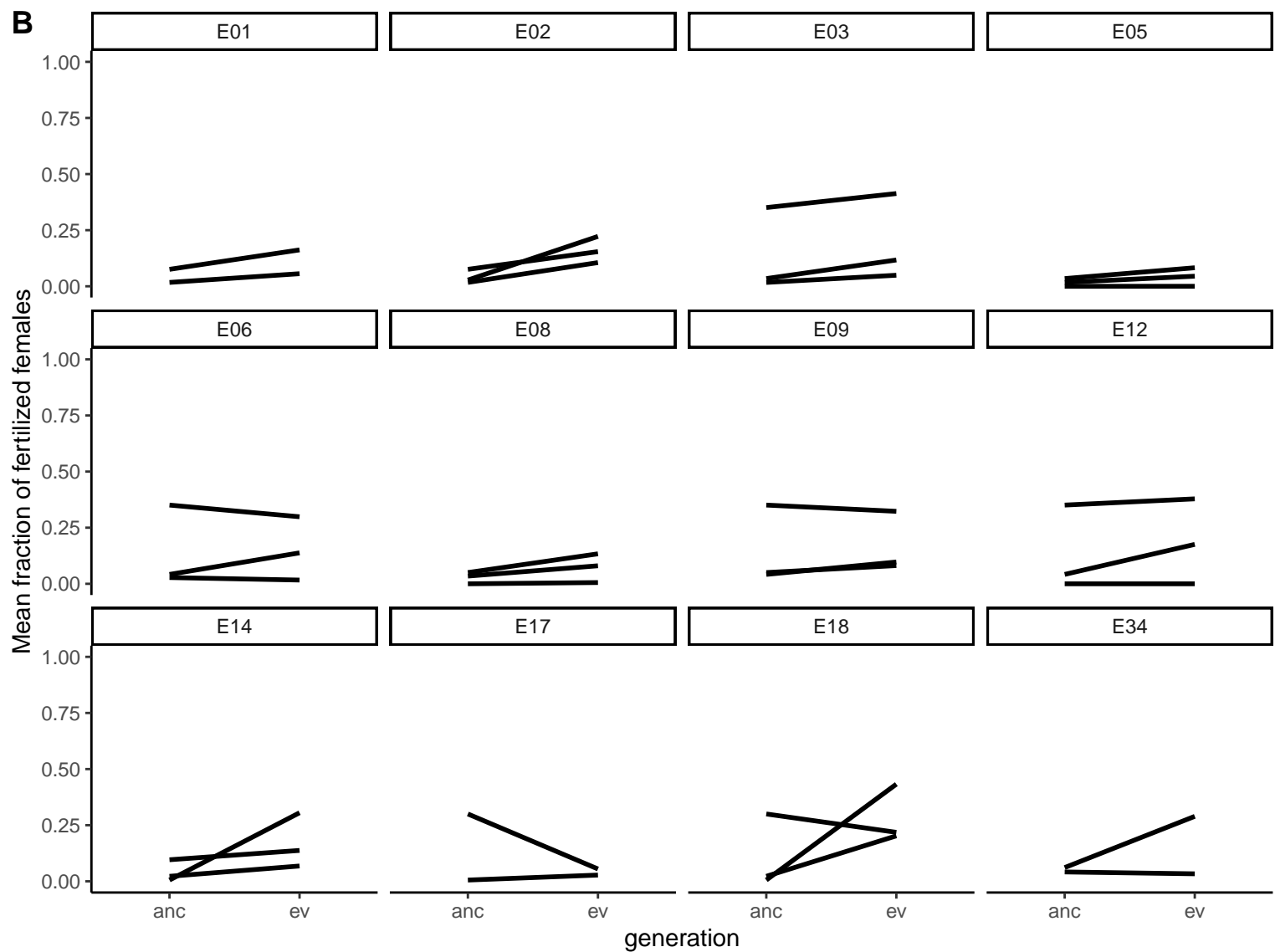

Supplement: Supplemental Information 2 — Populations from 20 °C are presented in panel A and from 24 °C are presented in panel B. Lines represent assay blocks. [file peerj-11-15825-s002.pdf]
